# Retraction: Design and synthesis of VEGFR-2 tyrosine kinase inhibitors as potential anticancer agents by virtual based screening

**DOI:** 10.1039/d5ra90070k

**Published:** 2025-06-04

**Authors:** Harun M. Patel, Pankaj Bari, Rajshekhar Karpoormath, Malleshappa Noolvi, Neeta Thapliyal, Sanjay Surana, Pritam Jain

**Affiliations:** a Dept. of Pharmaceutical Chemistry, University of KwaZulu-Natal (Westville Campus) Private Bag X54001 Durban-4000 South Africa; b Department of Pharmaceutical Chemistry, R.C. Patel College of Pharmacy Shirpur Dhule 425405 Maharashtra India pritash79@yahoo.com +91-2563-251808 +91-2563-255189; c Department of Pharmaceutical Chemistry, Shree Dhanvantary Pharmacy College Kim Surat-3941110 Gujarat India

## Abstract

Retraction of ‘Design and synthesis of VEGFR-2 tyrosine kinase inhibitors as potential anticancer agents by virtual based screening’ by Harun M. Patel *et al.*, *RSC Adv.*, 2015, **5**, 56724–56771, https://doi.org/10.1039/C5RA05277G.

The Royal Society of Chemistry hereby wholly retracts this *RSC Advances* article due to a significant number of errors in the data reported in the article.

In the Experimental section, with the exception of compound **41**, the reported yields and melting points of all compounds are incorrect.

The ^1^H NMR data reported in the Experimental section and the associated spectra in the supplementary information for compounds **26**, **29** and **55** are incorrect.

The HRMS data reported in the Experimental section and the associated spectra in the supplementary information for compounds **3**, **22**, **26**, **29**, **47**, **55** and **69** are incorrect.

In addition, all IC_50_ values in the article have been incorrectly reported as nM rather than μM.

The number and type of errors identified means that the Editor has lost confidence in the overall reliability of the article and therefore this paper is being retracted.

The authors were informed about the retraction. Harun M. Patel responded but did not state whether they agreed with the decision. The other authors have not responded.

Signed: Laura Fisher, Executive Editor, *RSC Advances*

Date: 20th May 2025

